# Engaging Rural Youth in Physical Activity Promotion Research in an After-School Setting

**Published:** 2005-10-15

**Authors:** Nancy O'Hara Tompkins, James A Rye, Sam Zizzi, Elizabeth Vitullo

**Affiliations:** Department of Community Medicine, West Virginia University; West Virginia University, Morgantown, WVa; West Virginia University, Morgantown, WVa; West Virginia University, Morgantown, WVa

## Abstract

**Background:**

West Virginia, the second most rural state in the nation, has a higher than average prevalence of chronic diseases, especially those related to physical inactivity and obesity. Innovative educational approaches are needed to increase physical activity among adults and youth in rural areas and reduce rural health disparities. This paper describes West Virginia's Health Sciences and Technology Academy (HSTA) Education and Outreach on Healthy Weight and Physical Activity. The project involved teachers and underserved high school students in social science research aimed at increasing physical activity among student and community participants.

**Context:**

The HSTA is an ongoing initiative of university–school–community partnerships in West Virginia that offers academic enrichment to high-school students in after-school clubs. For this project, six HSTA clubs were awarded grants to conduct research on physical activity promotion during the 2003–2004 school year. The project was funded by the Centers for Disease Control and Prevention.

**Methods:**

Focus groups, workshops, and targeted technical assistance were used to assist teachers and students with developing, implementing, and evaluating their research projects. Each club completed one project, and students reported on their research at the annual HSTA symposium held in the spring. Teachers documented their experience with the projects in process journals before and during implementation.

**Consequences:**

Data from the teachers' process journals revealed that they believed this research experience increased their students' interest in health and health science careers and increased their students' understanding of social science research methods. Challenges included lack of time after school to complete all activities, competing student activities, limited social science research experience of both teachers and students, and delays that resulted from a lengthy human subjects approval process.

**Interpretation:**

The entire process was too ambitious to be achieved in one school year. Recommendations for future implementation include offering training modules on social science research methods for both teachers and students. These modules could be offered as a graduate course for teachers and as an in-school elective within the curriculum or as a summer institute for students. This preparatory training might alleviate some of the time management issues experienced by all the projects and could result in more skilled teacher and student researchers.

## Background

Rural residents are more likely to be obese, smoke more, be less active, and have less healthy eating habits than residents of suburban areas (1). Patterson et al reported that obesity and physical inactivity are more common among rural than urban adults (2). West Virginia is the second most rural state in the nation. Lying in the heart of Appalachia, West Virginia has lower than average standards of living and educational attainment and a higher than average prevalence of chronic diseases, especially those related to physical inactivity, obesity, and smoking (3,4). Rural health disparities have been identified as an important focus for both researchers and practitioners (5,6). The need for innovative approaches to address obesity and physical inactivity in rural areas has been observed, as has the challenge to engage rural residents and practitioners in the process of community health improvement (2,7). Youth participation in community health improvement has also been recommended (8,9).

This paper describes a physical activity promotion project in West Virginia, the Health Sciences and Technology Academy (HSTA) Education and Outreach on Healthy Weight and Physical Activity. Funded by the Centers for Disease Control and Prevention (CDC), the project brought together university faculty, high school teachers, and high school students in the development, implementation, and evaluation of community-based interventions designed to promote physical activity. The project's objectives were to build the skills of teachers and students in social science research methods and to increase physical activity among school and community participants.

## Context

The HSTA is an ongoing initiative of university–school–community partnerships in West Virginia. The HSTA provides academic enrichment through summer institutes and after-school community-based clubs to over 700 high school students in the state. The HSTA also provides substantial professional development opportunities to high school teachers, who facilitate the academic enrichment experiences for students. The HSTA is governed at the community level by local boards comprised of community members and professionals and at the state level by a Joint Governing Board of community and university representatives (10,11).

HSTA clubs focus on building leadership skills, nurturing allegiance to home communities, and enhancing science and math competence, especially as it relates to human health. Hallmarks of the HSTA experience include opportunities for inquiry-based learning and Web authoring and for making presentations on projects at an annual symposium. HSTA clubs typically meet two times per month and conduct community service and research projects. Approximately 25% of participating students are African American, and almost one half are financially disadvantaged.

For this education and outreach endeavor, six HSTA clubs were awarded grants to carry out research projects to promote physical activity during the 2003–2004 school year. Each club submitted a grant proposal in the spring of 2003 through a Request for Proposals (RFP) process to a selection committee of professionals from the HSTA and the Center for Healthy Communities at West Virginia University. Information on components of the proposal is available from www.wv-hsta.org/cdc_chc/Grant_Proposal_Packet.htm The amount of each grant awarded was approximately $5000 and included a laptop computer and related software, digital camera, stipends or substitute teacher pay for attending workshops, and a copy and supply allowance of about $1400. One condition of the grant was that the clubs involve school and community partners, including one health professional. A university investigator was assigned to each club to provide overall support and guidance. Appropriate Institutional Review Board (IRB) approval was obtained from West Virginia University's Office of Research Compliance.

Of the six clubs awarded grants, four are in rural counties that have natural environments (mountainous) and built environments (rural roads with limited shoulders, lack of sidewalks) that pose significant challenges to a physically active lifestyle. Of the other two clubs, one is in an urban and one is in a semirural county. A recent analysis of county behavioral risk factor prevalence data showed that four of the counties were statistically significantly worse than the U.S. average for physical inactivity, and three were worse for obesity (3). One club is in a persistently economically distressed county, and two are in counties designated as at risk for being economically distressed (12). Lower income and limited economic opportunities in rural areas have been linked to rural areas having to choose between economic development and healthy environments (7).

## Methods

### Project development

**Focus groups**

Before proposals were submitted, two focus groups were held for HSTA teachers to ascertain their views on the problem of physical inactivity in West Virginia and to hear ideas they had for addressing the problem. Twenty-one teachers participated in the focus groups. Themes that emerged included the following:

- Economically disadvantaged populations are especially disadvantaged relative to physical activity opportunities.
- Schools and adjacent grounds should be centers or hubs for physical activity for the entire community; take advantage of facilities that already exist in the community.
- Social support is critical to increasing physical activity.
- Because of the many economic and physical barriers to physical activity, try a "do it on your own" approach, where participants plan for how they can increase their daily physical activity levels, including at home.

**Workshops**

Three workshops were provided to the teachers of funded clubs during the project development phase. Teachers received stipends for attending each workshop. The first two workshops provided technical assistance in fine-tuning their proposals, writing the IRB protocols, and training for ethics and the Health Insurance Portability and Accountability Act of 1996 (HIPAA)Three workshops were provided to the teachers of funded clubs during the project development phase. Teachers received stipends for attending each workshop. The first two workshops provided technical assistance in fine-tuning their proposals, writing the IRB protocols, and training for ethics and the Health Insurance Portability and Accountability Act of 1996 (HIPAA). Teachers were also trained to provide ethics and HIPAA training to all health professionals and students who would have contact with participants or participants' data. Each club submitted an "expedited" application, which required specification of 1) consent (for adult participants) or assent (for youth participants younger than 18 years of age) and, as needed, HIPAA authorization; 2) recruitment; and 3) data collection and management, including confidentiality. A university investigator was listed as the principal investigator, and the HSTA teachers were listed as co-investigators on the application.

The development, revisions, resubmission, and approval of each IRB protocol took 2 to 3 months. The need to train the HSTA students and other health professionals having contact with subjects or subjects' data about ethics and HIPAA was partially responsible for this lengthy approval process. An additional factor included the need to expand education and referral procedures for those projects collecting height and weight data from minors.

The third workshop focused on Web authoring and data analysis. Web page templates as well as a data entry protocol and associated spreadsheet templates were provided to the clubs.

**Process journals**

Teachers documented their experience with the projects in a process journal that they kept before and during implementation. The teachers' journals conveyed their responses to each phase of the process and were used to identify successes and challenges the clubs encountered in carrying out their research.

### Overview of projects

Approximately 65 students participated in the research projects. Each club developed its own project design, intervention strategies, timeline, and outcome measures. Information on each club's project is available from www.wv-hsta.org/cdc_chc/index.htm. Most clubs combined individually oriented strategies such as educational sessions with strategies designed to improve the social environment (e.g., buddy systems) and physical environment (e.g., signage and measurement for indoor and outdoor walking routes). All clubs used pedometers both for motivational and measurement purposes, as suggested by Tudor-Locke (13). Three clubs included the school nurse as a partner to collect height and weight data for the body mass index (BMI) calculation. Two of these clubs also collected percentage of body fat on adults using the Omron Body Fat Analyzer. Pedometer data were also included as outcome measures. 

Before implementation, students posed specific research questions related to their club's project to form the basis of the presentations at the annual HSTA symposium. The questions (Table) were related to the process of implementing the project or were derived from a specific set of pretest or posttest data.

### Project implementation

The HSTA clubs used a variety of participant recruitment strategies, including putting fliers in teachers' mailboxes and making announcements over the school's intercom system. Four of the six clubs met or exceeded their recruitment goals, and another club had to turn away potential participants. Once participants were recruited, students collected and entered their data.

Students in some clubs encouraged participants to increase steps through motivational messages left in teachers' mailboxes. One club invited a local exercise physiologist to lead one of the educational sessions. He focused on the *energy gap* concept coined by Hill et al and the importance of a physically active lifestyle to prevent heart disease (14). Another club provided resistive exercise bands to participants and facilitated a session led by a local occupational therapist. Another club created three color-coded indoor walking routes of differing lengths in their school and provided estimated corresponding energy expenditures. Throughout implementation, university partners kept in contact with the HSTA teachers by e-mail and telephone. Two issues of a project newsletter were published in spring 2004 to disseminate information among HSTA clubs about special features or events associated with the various projects.

Unfortunately, because of the late start of the projects, students had to enter the postproject data at the same time that they were preparing their presentations for the HSTA symposium. Therefore, most students did not use postproject data as originally planned, and the majority of the presentations were based on descriptive analyses. Approximately 35 student presentations were made about the HSTA physical activity promotion projects, and clubs continued to analyze data after the symposium.

## Consequences

### Teacher reflections

Teacher reflections from their preimplementation journals generally centered on getting their projects started. Gaining IRB approval was a relatively novel process to the teachers and was the most frequent preimplementation barrier reported by teachers. The need to gain approval before doing any recruiting surprised teachers. More importantly, teachers were concerned about the process taking so long that the interest of both students and community participants would wane. Supporting process journal excerpts include: "Downloading the initial IRB application was at best a shocking experience."

Related to IRB issues were concerns about the effort involved in collecting data on minors, which required assent forms, HIPAA forms for taking anthropometric measurements, or both. The effort needed to obtain the required certifications for ethics and HIPAA training by anyone, such as the school nurse, who would come in contact with subjects or subjects' data was another barrier expressed by one half of the teachers. One teacher wrote, "The constant juggling of my schedule . . . and everybody else who is involved has made this a [sic] far more difficult than I could have imagined."

Recognizing this barrier, the university partners gained approval from the university IRB for the HSTA students and others associated with the project to complete an alternative to the online ethics and HIPAA training. HSTA teachers provided training (based on an outline developed by university investigators), and the students and others in attendance subsequently signed a form stating that they had completed and would comply with the training (with special emphasis on confidentiality). In reflecting on this process, one teacher described the educational value of this approach:

We did both trainings as a whole group, which allowed for some group dynamics and discussion to take place as they debated some of the quiz answers. It was good to sit back and watch the students debate their point and cite specific reasoning or recall information they were presented it [sic] the module.

Another teacher suggested that the training stimulated interest in completing the online certification: "Students were very receptive to training. Several asked to get online and take the training. . . . They seemed to understand the importance and significance of the privacy issues."

Inclement weather and student attendance at HSTA club meetings were each cited as additional barriers by one half of the teachers. Although the overall nature of the preimplementation journal entries was more positive for some teachers (e.g., "We feel very encouraged that this will be a successful venture"), other teachers increasingly expressed concern about the workload, HSTA student commitment, and getting the project up and running in time. Related to this, it appeared that the HSTA students had little voice in the choice of topic for one of the projects.

Teachers devoted less of their writing during implementation to describing barriers, although some mentioned inclement weather and problems with the degree of HSTA student commitment: "Students lost interest by waiting so late in the year." Participant attendance and failure to return surveys were other barriers. Suggested solutions included offering participants shorter educational sessions and reducing the project implementation period "next year" from 4 months to 4 weeks. Most teachers' entries conveyed positive experiences for themselves, HSTA students, the project participants, or all three. One teacher was contacted by an area physician who was impressed with the club's efforts, which led to reflections about the need to continue their project another year. Another reflection spoke to the gardening project's impact on the general school curriculum and looked forward to next year: "Students [in regular science class] ask every day if they can go to the garden. . . . Next year the bed will be established, and I intend to try some different projects." 

One of the most positive teacher reflections speaks to the comprehensive impact of their project:

Overall, the students and I feel that the project was a great success. Many of the women are keeping up their pedometer use. . . . [W]e all learned a great deal about planning and carrying out a variety of different events and gained experience with collecting data. Most of all, we learned that an idea can grow into a project that can help others and make a difference in our community.

### Investigator reflections

For the investigators, the biggest challenges were lack of time and teachers' and students' limited experience in human subjects research methods. Teachers and students face many competing demands for their time after school. The proposal process was intended to ensure that only those teachers and clubs that were seriously interested in doing research would participate. In retrospect, it may have been better to employ a less time-consuming process, which may have resulted in getting the projects up and running more quickly.

The partnership piece of the projects also did not work as expected. Some clubs had difficulty finding partners, while other clubs underused their identified partners. There definitely was a need to better inform the local governing boards and increase their involvement; the clubs should have presented their proposed projects to the boards in an effort to generate enthusiasm and support and garner further partners for the project.

## Interpretation

Significant challenges were involved in engaging teachers and youth in this endeavor. Clearly, the research projects were too ambitious to undertake given the teachers' and students' limited human subjects research experience and the lack of time after school. Prospects for continuation of the projects as research endeavors are mixed. At the beginning of the new school year, teachers queried students about their interest in continuing the research projects. Most teachers reported student interest in continuing the projects as community service rather than research projects. The gardening project is being integrated into the school science curriculum.  

Other initiatives involving teachers and youth in research have reported similar challenges (9,15). The more successful initiatives provided ample opportunities for training and support of both teachers and students over longer periods of time. Perhaps these opportunities could be offered as a graduate course for teachers and as an elective in the school curriculum or at a summer institute for students. Distance education technologies might also be used, particularly for schools far away from universities. Consideration should also be given to doing the projects in phases, where needs assessment, project development, and IRB approval would occur in the first year and project implementation and evaluation in the second year. The first year also could include some pilot testing to ensure that HSTA students have ample input into project conception. We hope these lessons will enhance others' knowledge of the challenges associated with involving youth in health promotion research.

## Figures and Tables

**Table:** Sample Student Research Questions, Health Sciences and Technology Academy (HSTA) Education and Outreach on Healthy Weight and Physical Activity, West Virginia, 2003–2004

**Research Question**	**Data Source**	**Sample Size**	**Proposed Analysis**
What barriers to physical activity do participants report?	Barriers survey (preproject)	30	Descriptive statistics
Over a 10-week period, will the number of steps taken increase on a daily basis?	Daily step count from pedometer log	30	Descriptive statistics
Has being involved in this project changed the participants' concept of physical activity and influenced their future intentions concerning physical activity?	Survey (postproject)	20	Frequency and inductive analyses
Will our participants' scores on the barriers survey decrease from preassessment to the postassessment?	Barriers to physical activity survey (preproject and postproject)	35	Paired *t* test
At baseline, what percentage of participants are overweight and obese, and how do they compare with statewide BMI^a^ data from the West Virginia Behavioral Risk Factor Surveillance System?	BMI^a^ (preproject)	20	Descriptive statistics

a BMI indicates body mass index (kg/m^2^).
